# Efficacy of Simultaneous Administration of Nimodipine, Progesterone, and Magnesium Sulfate in Patients with Severe Traumatic Brain Injury: A Randomized Controlled Trial

**DOI:** 10.29252/beat-070206

**Published:** 2019-04

**Authors:** Ali Abdoli, Farshid Rahimi-Bashar, Saadat Torabian, Sepideh Sohrabi, Hamid Reza Makarchian

**Affiliations:** 1 *Department of Neurosurgery, Hamadan University of Medical Sciences, Hamadan, Iran *; 2 *Department of Anesthesiology and Critical Care, Hamadan University of Medical Sciences, Hamadan, Iran *; 3 *Department of Social Medicine, Hamadan University of Medical Sciences, Hamadan, Iran *; 4 *School of Medicine, Hamadan University of Medical Sciences, Hamadan, Iran *; 5 *Department of General Surgery, Hamadan University of Medical Sciences, Hamadan, Iran *

**Keywords:** Traumatic brain injury, Nimodipine, Progesterone, Magnesium sulfate

## Abstract

**Objective:**

To investigate the safety and efficacy of simultaneous administration of nimodipine, progesterone, magnesium sulfate in patients suffering from severe traumatic brain injury (TBI).

**Methods:**

Overall, 90 patients with blunt head trauma who were admitted to the Besat hospital, Hamadan University of Medical Sciences, Iran through the Emergency Department in 2017 to 2018 were randomly assigned to the study or control groups each containing 45 patients. In the study group, intravenous nimodipine 60 mg every 12 hours for 5 days, intramuscular progesterone 1 mg/kg daily for 5 days, and magnesium sulfate 5 grams stat followed by 2.5 grams every 4 hours for 21 days were administered. Daily GCS and jugular venous oxygen saturation (SjvO2) of the patients were measured on admission day (day 0) through hospitalization day 4 at the intensive care unit. Then, all patients were visited at three months after discharge.

**Results:**

The mean age of the patients was 31.4 ± 12.8 years including 59 (65.6%) men with no significant difference between the groups. The baseline GCS and SjvO2 of the patients were comparable in both groups, however, GCS of the patients in the study group were significantly higher in the next 4 hospitalization days compared to the controls. Whereas, the SjvO2 of the patients were not significantly different between the groups during these days. Three-month mortality rate of the patients in the study group was significantly lower than the three-month mortality rate of the patients in the control groups (22.2% *vs.* 42.2%, *p*=0.042).

**Conclusion:**

Administration of combined protocol of magnesium sulfide, progesterone and nimodipine may be safe and effective in patients suffering from severe TBI.

**Clinical Trial Registry:**

IRCT201210229534N2

## Introduction

Traumatic brain injury (TBI) is characterized as structural and physiological alterations in brain function caused by an external force [1]. It is a highly serious global health problem with incidence of 1.7 million of general population in the United States associated with about one third of trauma-related deaths [2]. Moreover, severe TBI which is classified as patients with TBI having the Glasgow Coma Scale (GCS) score of three to eight is responsible for 90% of all TBI related costs providing the importance of finding the underlying pathophysiology and management of severe TBI [3].

 Primary and secondary brain injury may result in significant and permanent disability. The primary traumatic brain injury is attributed to the mechanical force, applied to the brain tissue, however, subsequent to a primary insult, blood-brain barrier is disrupted, inflammatory cells are activated and recruited to the site causes oxidative stress, inflammation and apoptosis [4-6].

 Also, Disruption of neuronal metabolism due to vascular alterations results in oxidative stress, ion imbalance and neuronal death. The death of neurons may further damage nearby cells by releasing neurotoxic substances such as glutamate which excites neurons through (N-methyl-D-aspartate) NMDA receptors and cause intracellular calcium accumulation and calcium mediated apoptosis [7-9]. Secondary brain injury can be attenuated by interruption of this cascade especially in the time frame between primary and secondary brain injury [10]. Nimodipine, an L-type calcium channel blocker has been studied as a neuroprotective agent [11]. Influx of calcium is inhibited by nimodipine can cause neuroprotection through vasodilation and inhibition of intracellular calcium accumulation. Consequently, neuronal metabolism may be improved and calcium mediated apoptosis may be inhibited [12]. Magnesium as a noncompetitive inhibitor of NMDA receptor and antioxidant agent may play a role in inhibition of excitotoxic neuronal death and consequent attenuation of brain injury [13-15]. Moreover, progesterone has been shown to have neuroprotective activities through attenuation of inflammation and cerebral edema [16, 17]. However, in total, there is no consensus regarding an effective in attenuation of brain injury in TBI, since most of the agents either failed or showed minimal benefit in clinical trials [18-20]. Hence, in this study our aim is to find safety and efficacy of simultaneous administration of nimodipine, progesterone, magnesium sulfate in patients suffering from severe TBI. 

## Materials and Methods

 *Patients Selection*

 This randomized clinical trial was performed on patients with blunt head trauma who were admitted to the Besat hospital, Hamadan University of Medical Sciences, Iran through the Emergency Department in 2017-2018. Inclusion criteria were the patients with the diagnosis of severe TBI defined as GCS equal or less than 3 without concomitant injury. Exclusion criteria were patients with pregnancy, cardiac arrest, spinal cord injury, hemodynamic instability defined as hypovolemic shock, systolic blood pressure less than 90 mmHg requiring vasopressor drugs and prolonged hypoxemia defined as partial pressure of arterial O less than 60 mm Hg, need for cranial or other surgical interventions and on admission, history of estrogen or progesterone use in past 30 days and chronic kidney disease. All patients were evaluated in the Emergency Department by a same neurosurgeon. Patients by order of entry into the study were assigned consecutive numbers and were allocated to the study or control groups based on a predetermined computer-generated random list that only the trained critical care nurse was aware of. 

 *Study Protocol*

**Fig. 1:**
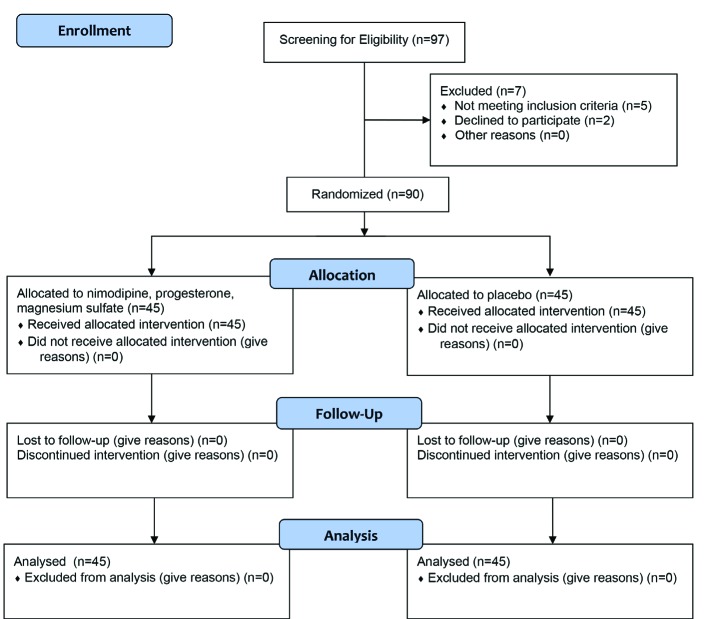
The CONSORT flow diagram of the study

 In the study group, intravenous nimodipine (Nimotop, Bayer Schering, Germany) at a dose of 60 mg every 12 hours for 5 days, intramuscular progesterone (Aburaihan Pharmaceutical Company, Iran) at a dose of 1 mg/kg daily for 5 days, and magnesium sulfate (Shahid Ghazi Pharmaceutical Co, Iran) at a dose of 5 grams stat followed by 2.5 grams every 4 hours for 21 days were administered by the same trained critical care nurse. All patients in both groups were given adequate intravenous fluid and same anticonvulsant, antacid and anticoagulant drugs. All were monitored closely and evaluated during the study by a same neurosurgeon who was not aware of the group of the patients. A central venous catheter was applied in jugular vein of all patients by which a 5 cc blood sample was taken daily tested for oxygen saturation. Daily GCS and jugular venous oxygen saturation (SjvO2) of the patients were measured on admission day (day 0) through hospitalization day 4 at the intensive care unit (Figure 1). The status of the patients were classified as death, vegetative state, severe disability, moderate disability, good recovery using Glasgow Outcome Scale (GOS) at the time of discharge as early GOS [21]. Then, all patients were visited by the same neurosurgeon with no information about the group of the patients at three months after discharge and their late GOS was recorded prospectively.

 *Ethics Approval*

 Our study was approved by the Ethics Committee of Hamadan University of Medical Sciences and Iranian Registry of Clinical Trials with reference number of IRCT201210229534N2. The study details and purposes were explained to legal representatives of our patients and written informed consent forms were taken.

 *Statistical Analyses* Data were analyzed using the SPSS statistical software (IBM SPSS Statistics for Windows, Version 23.0, IBM Corp., Armonk, NY, USA). Qualitative and quantitative data were compared using Chi-square and independent two sample-t tests, respectively. The results were considered statistically significant if *p *value was less than 0.05.

## Results

Ninety patients with the mean age of 31.4 ± 12.8 yrs/old including 59 (65.6%) males were randomly allocated to the study and control groups each containing 45 patients. The demographic data of the patients were not significantly different between the groups. In the study and control groups there were 28 and 31 males, respectively (*p*=0.51) and the means age of the patients were 29.3 ± 11.2 and 33.6 ± 14 yrs/old, respectively (*p*=0.11).

 The detailed outcome measurements in the study and control groups are shown in Table 1. The baseline GCS and SjvO2 of the patients were comparable in both groups however, GCS of the patients in the study group were significantly higher in the next 4 hospitalization days compared to the controls. Whereas, the SjvO2 of the patients were not significantly different between the groups during these days. Three-month mortality rate of the patients in the study group was significantly lower than the three-month mortality rate of the patients in the control groups (22.2% vs. 42.2%, *p*=0.042).

**Table 1:** Detailed outcome measurements in each groups

	**Study group** **(n=45)**	**Control group** **(n=45)**	***P*** **-value**
**GCS** a			
Day 0	7.64 ± 13.51	8.8 ± 15.38	0.706
Day 1	6.42 ± 1.32	5.8 ± 1.16	0.031
Day 2	6.84 ±1.47	6.06 ± 1.05	0.051
Day 3	7.6 ± 1.71	6.44 ± 1.5	0.001
Day 4	8.51 ± 1.89	6.64 ± 1.75	0.000
**SjvO2** b			
Day 0	77.15 ± 11.57	75.35 ± 11.25	0.457
Day 1	78.38 ± 10.85	75.86 ± 10.07	0.263
Day 2	78 ± 11.45	75.37 ± 10.10	0.253
Day 3	77.51 ± 10.67	76.97 ± 9.5	0.804
Day 4	77.9 ± 11.33	76.56 ± 8.72	0.545
**GOS** c			
Early	2.54 ± 0.5	2.73 ± 0.45	0.139
Late	1.4 ± 0.6	1.84 ± 0.97	0.031

a GCS, Glasgow coma scale;

b SjvO2, jugular venous oxygen saturation;

c GOS, Glasgow outcome scale; Data are presented as mean ± standard deviation

## Discussion

In TBI the primary event is not improvable, whereas the secondary cascade is theoretically amenable to treatment [18]. The effort is being made to minimize secondary brain damage and to provide the best chances of recovery after the initial injury. However, there are currently no pharmacologic treatments that have unequivocally been proven to protect against these detrimental consequences of TBI [10, 18]. The overall neurologic recovery and long-term morbidity remain to represent a significant healthcare challenge [2, 3].

A dihydropyridine-derived calcium antagonist so-called nimodipine is highly lipophilic, it easily pass through blood-brain barrier and can cause selective cerebral arterioles vasodilation without significant systemic hypotension [22]. In addition to attenuating vasospasm and improving blood flow in process of TBI, regulating calcium hemostasis and oxidative stress can significantly change the course of TBI’s deleterious cascade [7, 18]. In a systematic review of randomized controlled trials of calcium channel blockers in acute traumatic head injury patients shows that significant ambiguity remains over their effects [23]. The effect of nimodipine in a subgroup of brain injury patients with subarachnoid hemorrhage shows a beneficial effect, though the increase in adverse events suffered by the intervention group may warn cautious use of this drug in patients [24]. In study by Farhoudi *et al*., 40 patients suffered from diffuse axonal injury with GCS of 5-8 were randomly treated with 60mg of nimodipine every 4 hours immediately after admission. No superior outcome in prognosis was seen [25]. However, Aslan et al. treated 5 patients with 1mg/h in the first 2 h, and 2mg/h for the rest of the Hours for a week by nimodipine. It was revealed that nimodipine can improve cerebral metabolism, jugular venous oxygen saturation and outcome in patient with severe head trauma [12]. Antioxidative role of nimodipine has also been shown in severe head trauma by Aslan *et al*., [26]. 

Progesterone has been shown to have pleiotropic neuroprotective properties. Multiple animal studies with variety of methods has confirmed the benefit of progesterone in TBI. Multifactorial effects of progesterone include inflammation reduction, inhibition of inflammatory cytokines, apoptosis reduction, prevention of excitotoxicity and lessening edema. The progesterone receptor plays a key role in these neuroprotective effects [16, 17]. However, human studies has shown mixed results [27, 28].  In two large clinical trials no benefit were shown by progesterone treatment in patients with either moderate to severe or severe TBI [29, 30]. 

Magnesium ion is an abundant intracellular cation, plays a vital role in cellular metabolism. It interrupts a number of secondary factors involved in pathophysiology of TBI, the principal action that has been suggested by animal models is by NMDA receptor blockade and decreasing glutamate release. The other neuroprotective mechanisms proposed are improvement of cerebral blood flow, calcium channel blockage and inhibition of apoptosis [15, 31, 32]. A recent meta-analysis of existing randomized controlled trials did not identify a significant beneficial effect in the mortality of traumatic brain injury patients; however, it suggests that magnesium sulfate shows a tendency to improve the GOS and GCS scores, which is a promising result for traumatic brain injury therapy [33]. In the present study, a combination of drugs was administered to improve patient outcome. GCS was significant improved after 24 hours and remained significantly better than control group at discharge. Although GOS was not significantly different at discharge, the improvement after three months was significantly superior to control group. Also, significantly lower mortality was seen in treatment group. 

SjvO2 measurement offers evidence regarding the balance between supply and demand of brain oxygen status. Unemployed oxygen in the brain is transported to the systemic circulation through internal jugular vein. Therefore, SjvO2 measurement can determine the balance between cerebral metabolic requirement of oxygen and cerebral blood flow.  Normal value of SjvO2 is 55–75%. Various pathologies can either decrease or increase the SjvO2. Vasospasm, hyperventilation, fever, seizure, reduced cerebral perfusion pressure and increased cerebral metabolic requirement of oxygen are among causes to decrease SjvO2 values. On the other hand, decreased cerebral metabolism requirement (due to cell death or mitochondrial dysfunction), hyperemia condition, and microvascular shunting due to oxygen extraction and diffusion disturbance on the damaged brain tissue can increase SjvO2 [34, 35]. SjvO2 in TBI has been shown to be a determinant in outcome. Previously, strong positive correlation between SjvO2 and prognosis and GCS was shown [36, 37], similarly, negative correlation with Full Outline of Responsiveness score has been revealed [35]. Although Aslan *et al*. showed an increase in SjvO2 in nimodipine treated group, in the presented study no statistically significant difference was observed in control and treatment group [12]. 

In conclusion, administration of combined protocol of magnesium sulfide, progesterone and nimodipine may be safe and effective in patients suffering from severe TBI. Further, large, multicenter studies are required to shed light on the issue. 

## Conflict of Interest:

None declared.
